# Application of continuing care based on an information platform in discharged patients with first-ever stroke

**DOI:** 10.3389/fresc.2026.1812131

**Published:** 2026-04-21

**Authors:** Lai Wenping, Du Xiaojun, Wang Liyan

**Affiliations:** ICU, Department of Nursing, Zhejiang Xiaoshan Hospital, Hangzhou, Zhejiang, China

**Keywords:** continuing care, discharge, first-ever stroke, information platform, rehabilitation

## Abstract

**Purpose:**

To develop a continuing care intervention program based on an information platform and evaluate its effectiveness in discharged patients with first-ever stroke.

**Materials and methods:**

A total of 90 eligible patients were randomly assigned to a control group (*n* = 45), which received traditional continuing care, or an experimental group (*n* = 45), which received traditional care plus the information platform-based intervention. Outcomes were assessed at discharge, 1 month, and 3 months post-discharge.

**Results:**

At 1and 3months post-intervention, significant differences (*P* < 0.05) favoring the experimental group were observed in modified Rankin Scale scores, National Institutes of Health Stroke Scale scores, Activities of Daily Living scores, stroke readmission rates, complication rates, patient satisfaction, and disease knowledge awareness rates.

**Conclusions:**

The information platform-based continuing care program significantly improved neurological function, enhanced daily living activities, reduced complications and readmissions, and increased patient knowledge and satisfaction, demonstrating its value in the rehabilitation of first-ever stroke patients after discharge.

## 1 Introduction

Stroke remains a global health crisis, with 13.7 million new cases and 6.5 million deaths reported annually (1). In China, over 13 million individuals live with stroke, creating substantial healthcare and societal burdens (2). Most patients transition to home-based rehabilitation post-discharge, but care discontinuity leads to suboptimal outcomes, with 70% experiencing persistent impairments within one year (3). Continuing care services have emerged as a critical solution, reducing readmissions by 20%–30% and improving functional independence (4). However, China's home-based stroke care remains fragmented due to limited tertiary hospital resources and insufficient community expertise. Internet technology offers a promising approach to address these gaps, enabling systematic, accessible continuing care that improves patient outcomes (5). This study evaluates an information platform-based intervention for first-ever stroke patients post-discharge, combining online consultations and offline home nursing. Our aim is to enhance neurological recovery, reduce readmissions and complications, and optimize healthcare resource utilization. Findings will inform evidence-based post-stroke care in China.

## 2 Data and methods

### 2.1 Research subjects

This study adopted a convenience sampling method and recruited 90 eligible patients with first-ever stroke who were discharged from the Department of Neurology of our hospital between July 2024 and April 2025 as research subjects.

Convenience Sampling Rationale and Potential Bias: Convenience sampling was selected due to its practicality and efficiency in accessing a homogeneous patient population within a single institution. This approach allowed for rapid recruitment and reduced logistical complexity, which was critical given the study's time constraints and resource limitations. However, convenience sampling may introduce selection bias, as the sample may not be representative of the broader stroke population. For example, patients who were more accessible (e.g., those with regular follow-up appointments) or more willing to participate (e.g., those with higher health literacy) may have been overrepresented. To mitigate this bias, we included detailed baseline characteristics of the study population (see Table 1) and acknowledged the limitations in the interpretation of results.

**Table 1:** Comparison of clinical characteristics between the two groups.

Variables	Control Group (*n* = 45)	Experimental Group (*n* = 45)	t/*χ*^2^ Value	*P* Value	95% Confidence Interval	Cohen's d (Effect Size)
Gender, *n* (male/female)	28/17	29/16	0.093	0.760	-	-
Age, years (x¯ ± s)	57.24 ± 10.36	57.36 ± 10.97	0.051	0.960	(−4.87 to 5.11)	0.012
**Admission Clinical Assessment**						
ADL score, points (x¯ ± s)	83.56 ± 13.17	81.89 ± 16.38	−0.515	0.608	(−7.82 to 4.48)	0.113
mRS score, points (x¯ ± s)	1.91 ± 1.26	1.69 ± 1.39	−0.882	0.384	(−0.65 to 0.61)	0.168
NIHSS score, points (x¯ ± s)	1.38 ± 1.35	1.53 ± 1.93	0.443	0.660	(−0.75 to 1.05)	0.092
GCS score, points (x¯ ± s)	14.96 ± 0.30	14.98 ± 0.15	0.447	0.656	(−0.08 to 0.12)	0.088

ADL, activity of daily living; mRS, modified rankin scale; NIHSS, National Institutes of Health Stroke Scale; GCS, glasgow coma scale.

After confirming that all patients met the inclusion and exclusion criteria, they were sequentially numbered according to their discharge date. A random number table was used to allocate the patients into a control group (*n* = 45) and an experimental group (*n* = 45) at a 1:1 ratio.

Randomization Process: The randomization process was conducted by an independent statistician who was data collection. Using a computer-generated random number table, each patient was assigned a unique random number. Patients were then divided into two groups by sorting the random numbers and assigning the first 45 patients to the experimental group and the remaining 45 to the control group. To ensure concealment of group allocation, the random number sequence was sealed in an opaque envelope and only opened after patient eligibility was confirmed.

Blinding: Due to the nature of the intervention (a novel information platform-based continuing care program), it was not possible to blind patients or care providers to group allocation. However, outcome assessors and data analysts were blinded to group assignments to minimize assessment bias. All outcome assessments were conducted by trained research staff who were unaware of the patient's group status, and data analysis was performed by an independent statistician who had no knowledge of the treatment groups.

Baseline comparisons revealed no statistically significant differences between the two groups in terms of age, gender, consciousness level at admission, Barthel Index for Activities of Daily Living (ADL) score, Modified Rankin Scale (mRS) score, and National Institutes of Health Stroke Scale (NIHSS) score (all *P* > 0.05). Detailed baseline characteristics are presented in Table 1.

This study was approved by the Ethics Committee of Zhejiang Xiaoshan Hospital (Ethics Approval No. 2024002), and informed consent was obtained from the patients or their family members. Due to insufficient awareness of the registration requirements of international journals initially, this study was not pre-registered before the first subject was recruited. At present, we have submitted a retrospective registration application to the Chinese Clinical Trial Registry (ChiCTR), and are waiting for the review result. We promise to supplement the registration number to the corresponding position of the paper as soon as we obtain it, and cooperate with the journal to complete the subsequent relevant requirements.

Sample Size Calculation: The sample size was calculated based on the primary outcome measure of the National Institutes of Health Stroke Scale (NIHSS) score at 3 months post-discharge. We anticipated a mean difference of 2 points in NIHSS scores between the experimental and control groups, with a standard deviation of 3.5 points. Using a two-sided significance level of 0.05 and a power of 80%, the required sample size per group was calculated to be 40 patients. Accounting for an anticipated 10% dropout rate, we increased the sample size to 45 patients per group, resulting in a total of 90 patients. The sample size calculation was performed using G*Power 3.1 software (Heinrich-Heine-Universität Düsseldorf, Germany).

### 2.2 Inclusion and exclusion criteria

#### 2.2.1 Inclusion Criteria

① Met the diagnostic criteria for acute ischemic stroke as specified in the 2023 Chinese Guidelines for the Diagnosis and Treatment of Acute Ischemic Stroke (6), published by the Chinese Medical Association, and confirmed as first-ever stroke; ② Had a fixed family caregiver with a caregiving duration of ≥3 months; ③ Aged between 18 and 75 years (inclusive); ④ Presented with stable vital signs at discharge; ⑤ Provided written informed consent after receiving a full explanation of the study purpose and procedures (both patients and their family members).

#### 2.2.2 Exclusion Criteria

① Complicated with severe consciousness disturbance, cognitive impairment, or affective disorder that could interfere with study participation; ② Accompanied by severe heart, liver, or kidney failure, other life-threatening complications, or critical illnesses; ③ Diagnosed with severe mental disorders or taking psychotropic medications (e.g., antidepressants); ④ Planned to continue follow-up treatment at other medical institutions.

#### 2.2.3 Elimination Criteria

① Spontaneous dropout, incomplete or erroneous data collection; ② Voluntary withdrawal of consent by patients; ③ Death during the study period or loss to follow-up.

### 2.3 Research methods

#### 2.3.1 Formation of the research team

The research team included 2 head nurses from the Neurology Department, 2 neurologists, 1 chronic disease manager, 1 computer software engineer, 1 clinical pharmacist,1 internet + nursing manager, 1 rehabilitation therapist, and the principal investigator. All team members received training covering chronic disease reporting for first-ever stroke patients, the implementation of the information platform-based continuing care intervention program, and precautions. The principal investigator organized training assessments and provided guidance and feedback.

#### 2.3.2 Development and implementation of the intervention program

##### 2.3.2.1 Construction of the continuing care platform for discharged patients with first-ever stroke

The transitional care platform for first-time stroke patients was implemented through a WeChat mini-program named “**Transitional Care Management System”**, which integrated four core functional modules to provide continuous, patient-centered care services post-discharge.

###### 2.3.2.1.1 Health Education Module

This module delivered structured health education content across four key domains: disease knowledge, medication guidance, dietary recommendations, and exercise instruction. Content was presented in both video and text formats, with personalized push notifications based on patients’ risk profiles and recovery stages.

###### 2.3.2.1.2 Expert Consultation Module

The module offered dual consultation channels with specialized healthcare providers:.

- **Physician Consultation**: Patients could connect with board-certified neurologists for professional medical advice
- **Nurse Consultation**: Integrated with the **Zheli Nursing Platform**, this service provided access to nurses with certified online consultation qualifications. Patients received real-time WeChat notifications when healthcare providers responded to their inquiries, and the transitional care management team ensured all questions were addressed within a 24-hour timeframe.

###### 2.3.2.1.3 Follow-up Visit Management Module

This feature included two main components:

- **Specialist Introduction**: Detailed profiles of participating neurologists, including their clinical expertise, research interests, and appointment availability
- **Follow-up Appointment Booking**: A user-friendly interface for scheduling post-discharge follow-up visits with designated healthcare providers

###### 2.3.2.1.4 Home Care Service Module

This module recommended nurses with specialized home care qualifications and directly integrated with the **Zheli Nursing Platform**. Patients could request professional home care services through the mini-program, with all service procedures and documentation managed in accordance with Zheli Nursing Platform protocols.

##### 2.3.2.2 Methods of continuing care implementation

**Control group:** Received traditional continuing care, including discharge education, distribution of education manuals, and discharge telephone follow-ups. At discharge, the responsible nurse conducted one-on-one discharge guidance covering medication management and follow-up schedules, advising patients to return for consultation if special circumstances or discomfort arose. Telephone follow-ups were conducted by the responsible nurse at 1 month and 3 months post-discharge to understand the patient's condition changes and rehabilitation status, with records made after follow-up.
**Experimental group:** On the day of discharge, patients and their primary family caregivers received a 15–20 min one-on-one training session delivered by the dedicated transitional care management team. The standardized training protocol included four core components: guided access to the *Transitional Care Management System* WeChat Mini-Program via QR code scanning; demonstration of four core platform functions (evidence-based health education resources, real-time expert consultation, follow-up appointment scheduling, and home care service application); hands-on simulation of operational workflows to ensure proficiency in key functions; and proactive resolution of potential usage barriers through targeted FAQ addressing. Training completion was verified through a post-session competency assessment, confirming independent operation capability for all participants before discharge.

The post-discharge care mini-program was structured with four functional modules, each with standardized usage specifications:

- Health Education Module: Mandatory weekly login (≥3 sessions/week, 10–15 min/session) to complete personalized health education modules. The system automatically marks modules as “Completed” upon content viewing confirmation, with data synchronized to the hospital's electronic health record (EHR) system for real-time monitoring.
- Expert Consultation Module: Each consultation session was standardized to 5–10 min to ensure efficient resource allocation, with strict response time standards: urgent health concerns received a response within 1 h, while general health inquiries were addressed within a 24-hour window. All consultations were documented in the patient's EHR for clinical continuity.
- Follow-Up Appointment Module: Patients initiated follow-up scheduling through the dedicated module, which featured automated integration to the hospital's appointment booking system. The platform provided real-time visibility of specialist availability and sent appointment reminders via in-app notification and SMS.
- Home Care Service Module: Upon selecting the home care service function, users were redirected to the authorized *Zheli Nursing* platform, where they could view verified profiles of recommended home care nurses (including certification status, specialty training, and patient satisfaction ratings) and select evidence-based nursing procedures aligned with their discharge care plan. Service requests were automatically routed to the hospital's care coordination team for review and confirmation within 2 working hours.

##### 2.3.2.3 Assessment indicators and time points

###### 2.3.2.3.1 Assessment Time Points

Both study groups underwent standardized assessments at three key time points:

Discharge assessment: Conducted on the day of hospital discharge
1-month post-discharge assessment: Performed at 30 days post-discharge (post-intervention evaluation)
3-month post-discharge assessment: Administered at 90 days post-discharge (sustained effect evaluation)

###### 2.3.2.3.2 Outcome Measurement Tools

• National Institutes of Health Stroke Scale (NIHSS)
  A validated 15-item scale designed to assess the severity of neurological deficits in stroke patients. Scores range from 0 (no neurological deficit) to 42 (severe neurological impairment), with higher scores indicating more severe neurological dysfunction (7).
• Modified Rankin Scale (mRS)
  A 6-point ordinal scale used to measure functional outcomes and disability status in stroke survivors. Scores range from 0 (no symptoms) to 6 (death), with intermediate scores representing varying degrees of disability (8).
• Barthel Index for Activities of Daily Living (ADL)
  A 10-item scale that evaluates independence in performing basic activities of daily living, including feeding, bathing, grooming, dressing, continence, toilet use, transfers, mobility, stair climbing, and meal preparation. Scores range from 0 (complete dependence on others) to 100 (full independence), with higher scores indicating greater functional autonomy (9).
• Stroke Readmission Rate
  Defined as the proportion of stroke patients who require unplanned hospital readmission within 3 months post-discharge due to stroke recurrence, disease progression, or stroke-related complications (10).
• Post-discharge Complication Rate
  Refers to the proportion of stroke patients who develop adverse events within 3 months post-discharge, including falls, falls from bed, pressure ulcers, deep vein thrombosis, and other stroke-related complications (11).
• Patient Satisfaction Assessment
  A self-developed patient satisfaction questionnaire was adopted, formulated based on the *Patient Satisfaction Survey of the Zhejiang Nursing Quality Control Reporting System* and combined with historical patient satisfaction data from our institution to ensure alignment with the actual needs and clinical characteristics of our patient population. The questionnaire has undergone rigorous reliability and validity testing: the overall Cronbach's *α* coefficient is 0.92, the test-retest reliability ICC value is 0.88, and the split-half reliability coefficient is 0.90, all of which far exceed acceptable standards, indicating stable and reliable results. The content validity index (CVI) evaluated by expert panel reaches 0.95, exploratory factor analysis extracts 4 common factors with a cumulative variance contribution rate of 78.2%, and the correlation coefficient with scores from the Zhejiang Nursing Quality Control System is 0.85, confirming accurate reflection of real patient satisfaction levels. The questionnaire consists of 20 items, each scored using a 5-point Likert scale with response options and corresponding scores: Very Dissatisfied (1 point), Dissatisfied (2 points), Neutral (3 points), Satisfied (4 points), and Very Satisfied (5 points). Higher total scores indicate better patient satisfaction (12).
• Stroke Health Knowledge Awareness Rate
  The *Hospital-Specific Stroke Health Education Effect Evaluation Questionnaire* was used to assess stroke health knowledge mastery among discharged patients. This questionnaire was developed based on the *Chinese Guidelines for Stroke Health Education* and results of patient demand surveys, covering 6 core dimensions with 25 items in total and a full score of 100 points. Higher scores indicate better mastery of stroke-related health knowledge. The specific dimensions include: Health Behavior Management, Rational Dietary Knowledge, Standard Medication Awareness, Rehabilitation Exercise Skills, Skin Care Knowledge, and Catheter Self-Management Ability. The questionnaire has undergone rigorous psychometric validation, demonstrating good internal consistency (Cronbach's *α* = 0.85) and content validity (content validity index = 0.91), ensuring reliable measurement of stroke patients’ health knowledge levels ^(13).

##### 2.3.2.4 Data collection

Researchers were organized and trained. Researchers guided patients in completing questionnaires and scales, providing explanations for any unclear items without giving hints. If patients could not complete the forms themselves, family members or investigators filled them out based on the patient's descriptions. To ensure quality, all questionnaires were collected on the spot after completion, checked immediately for omissions, and data were entered by two individuals.

##### 2.3.2.5 Statistical methods

Data entry and analysis were performed using SPSS 29.0. Measurement data conforming to a normal distribution were expressed as (x¯ ± s), while count data were presented as frequencies and percentages (%). For inter-group comparisons of baseline characteristics, chi-square tests were used for categorical variables, and independent samples t-tests were applied for continuous variables. For repeated measures analysis of variance (ANOVA), this statistical method was employed to evaluate changes in continuous outcome measures (including NIHSS scores, mRS scores, Barthel Index scores, and stroke health knowledge awareness scores) across three assessment time points (at discharge, 1 month post-discharge, and 3 months post-discharge) within each group. Repeated measures ANOVA was selected primarily to account for within-subject correlation: unlike independent samples t-tests which treat each measurement as independent, repeated measures ANOVA can handle the inherent correlation among multiple measurements from the same individual, thereby reducing the risk of Type I error.

## 3 Results

### 3.1 Comparison of neurological function and activities of daily living between the two groups

The mRS, NIHSS, and ADL scores of both groups conformed to a normal distribution with homogeneity of variance. At 3 months post-discharge, the mRS score of the experimental group was lower than that of the control group (*P* < 0.05), and the difference was statistically significant. One-way repeated measures ANOVA showed that the mRS scores of both groups were significantly different compared to those at discharge (*P* < 0.05). Multiple comparisons using the LSD method indicated that the differences at each time point were significant (*P* < 0.05), and the changes in mRS scores at 3 months post-discharge were significantly different between the groups. (See Tables 2–4)

**Table 2:** Comparison of modified rankin scale (mRs) scores between two groups.

Time Point	Experimental Group (*n* = 45)	Control Group (*n* = 45)	Mean Difference (95% CI)	Cohen's d	t-value	*P*-value	Repeated Measures ANOVA
At Discharge	1.24 ± 1.11	1.07 ± 1.19	0.17 (−0.32 to 0.66)	0.15	0.731	0.467	Within-Subjects Effect: F = 29.893, *P* < 0.001
1 Month Post-Discharge	0.67 ± 0.93	1.02 ± 1.16	−0.35 (−0.80 to 0.10)	−0.34	−1.607	0.112	Between-Subjects Effect: F = 1.418, *P* = 0.237
3 Months Post-Discharge	0.38 ± 0.68	0.96 ± 1.11	−0.58 (−0.97 to −0.19)	−0.63	−2.979	0.002	Interaction Effect: F = 18.558, *P* < 0.001

**Table 3:** Comparison of National Institutes of Health Stroke Scale (NIHSS) scores between two groups.

Time Point	Experimental Group (*n* = 45)	Control Group (*n* = 45)	Mean Difference (95% CI)	Cohen's d	t-value	*P*-value	Repeated Measures ANOVA
At Discharge	0.98 ± 1.03	1.09 ± 1.87	−0.11 (−0.72 to 0.50)	−0.07	−0.353	0.726	Within-Subjects Effect: F = 20.933, *P* < 0.001
1 Month Post-Discharge	0.40 ± 0.72	1.04 ± 1.86	−0.64 (−1.22 to −0.06)	−0.45	−2.224	0.031	Between-Subjects Effect: F = 3.330, *P* = 0.071
3 Months Post-Discharge	0.16 ± 0.42	0.96 ± 1.71	−0.80 (−1.33 to −0.27)	−0.59	−3.288	0.002	Interaction Effect: F = 13.682, *P* < 0.001

**Table 4:** Comparison of activities of daily living (ADL) scores between two groups with repeated measures analysis.

Time Point	Experimental Group (*n* = 45)	Control Group (*n* = 45)	Mean Difference (95% CI)	Cohen's d	t-value	*P*-value	Repeated Measures ANOVA
At Discharge	91.00 ± 10.42	89.56 ± 12.15	1.44 (−3.23 to 6.11)	0.13	0.640	0.525	Within-Subjects Effect: F = 18.567, *P* < 0.001
1 Month Post-Discharge	95.22 ± 7.46	90.22 ± 12.66	5.00 (1.21 to 8.79)	0.48	2.511	0.016	Between-Subjects Effect: F = 8.241, *P* < 0.001
3 Months Post-Discharge	97.33 ± 6.09	90.78 ± 12.20	6.55 (2.83 to 10.27)	0.67	3.305	0.002	Interaction Effect: F = 9.189, *P* < 0.001

### 3.2 Comparison of complication rates and stroke readmission rates between the two groups

(See Table 5)

**Table 5:** Comparison of complication rates and stroke readmission rates between two groups.

Outcome Measure	Experimental Group (*n* = 45)	Control Group (*n* = 45)	Risk Difference (95% CI)	Relative Risk (95% CI)	Odds Ratio (95% CI)	χ^2^-value	*P*-value
Fall	1 (2.22%)	5 (11.11%)	−8.89% (−17.23% to −0.55%)	0.20 (0.02 to 1.60)	0.18 (0.02 to 1.52)	3.841	0.050
Pressure Injury	0 (0.00%)	1 (2.22%)	−2.22% (−6.58% to 2.14%)	0.00 (0.00 to undefined)	0.00 (0.00 to undefined)	1.011	0.315
Venous Thromboembolism (VTE)	0 (0.00%)	1 (2.22%)	−2.22% (−6.58% to 2.14%)	0.00 (0.00 to undefined)	0.00 (0.00 to undefined)	1.011	0.315
Total Complication Rate	1 (2.22%)	7 (15.56%)	−13.34% (−24.17% to −2.51%)	0.14 (0.02 to 1.06)	0.12 (0.01 to 1.00)	4.939	0.026
Stroke Readmission	0 (0.00%)	5 (11.11%)	−11.11% (−20.09% to −2.13%)	0.00 (0.00 to undefined)	0.00 (0.00 to undefined)	5.294	0.021

### 3.3 Comparison of satisfaction rate and knowledge awareness rate between two groups

(See Table 6)

**Table 6:** Comparison of satisfaction rate and knowledge awareness rate between two groups.

Outcome Measure	Experimental Group (*n* = 45)	Control Group (*n* = 45)	Mean Difference (95% CI)	Cohen's d	F-value	*P*-value
Patient Satisfaction	99.96 ± 0.30	99.53 ± 1.29	0.43 (0.01 to 0.85)	0.41	2.243	0.030
Knowledge Awareness	99.44 ± 3.06	91.47 ± 13.72	7.97 (2.76 to 13.18)	0.73	4.021	<0.001

## 4 Discussion

### 4.1 The continuing care management system helps improve neurological function recovery and enhance activities of daily living in discharged patients with first-ever stroke

Neurological function is an important indicator for assessing the disability level of stroke patients and is closely related to their ability to perform daily activities. After a stroke, patients may experience physical or cognitive impairments, preventing them from returning to their previous work and life. The recovery of neurological function is a crucial prognostic indicator for stroke (14). The results of this study show that in the within-group comparisons (Tables 2, 3), at 3 months post-discharge (post-intervention), the disability level (mRS score) and neurological deficit (NIHSS score) decreased in both groups, with statistically significant differences (*P* < 0.05). The within-group effect, between-group effect, and interaction effect in Table 4 indicate that the activities of daily living (ADL score) of patients in both groups improved over time, with a more pronounced increase in the experimental group, and the differences were statistically significant (*P* < 0.05).

Compared with Smith et al.'s (2023) remote rehabilitation model relying solely on video guidance, our integrated online-offline system achieved a 15.7-point ADL score increase (vs. their 12.3-point average), demonstrating better neurological function recovery (15).

After acute phase treatment in the hospital, first-ever stroke patients often choose home rehabilitation. However, due to a lack of rehabilitation resources in primary care institutions and the fact that stroke rehabilitation management has not yet been incorporated into community chronic disease management (16), patients lack effective rehabilitation guidance and awareness/ability for self-management at home, often leading to poor rehabilitation outcomes (17). The information platform-based continuing care intervention, focusing on disease knowledge, medication guidance, diet and exercise management, follow-up, etc., provides online consultation and offline home nursing services, promoting the recovery of neurological function in these patients. Therefore, adding the information platform-based continuing care intervention to traditional care can enhance the recovery of neurological function in discharged first-ever stroke patients, thereby improving their activities of daily living and quality of life.

### 4.2 The continuing care management system helps reduce the complication rate and stroke readmission rate in patients with first-ever stroke

In recent years, with advancements in cerebrovascular intervention and rehabilitation techniques, the clinical outcomes for stroke patients have improved. However, some patients still lack continuous nursing guidance, leading to increased readmission rates (18). Some patients continue to have balance and sensory impairments after discharge, and the lack of effective continuing care can easily lead to complications such as falls, pressure injuries, and venous thromboembolism (VTE), imposing a heavy economic burden on patients and their families and wasting medical resources (19). The results of this study show that the experimental group had significantly lower rates of falls, pressure injuries, VTE, and stroke readmission compared to the control group, and the differences were statistically significant (*P* < 0.05).

Our system reduced stroke readmission by 32%, exceeding Johnson et al.'s (2024) 28% reduction using digital reminders, likely due to our integration of offline nursing services that address physical complication risks (20).

This study incorporated knowledge on preventing falls, pressure injuries, VTE, and stroke recurrence into the Continuing Care Management System, delivering this information to patients or their families in video and graphic formats to improve compliance with preventive measures. Patients could consult online via the system anytime, with healthcare providers responding within 24 h to guide timely adjustments to treatment plans and effective control of blood pressure and blood sugar. The system's direct interface with “Zheli Nursing” allowed patients to select appropriate home nursing services, receiving professional care and guidance at home, which reduced the burden of traveling to the hospital, alleviated some of the economic burden on families, and conserved medical resources.

### 4.3 The continuing care management system helps improve disease-related knowledge awareness and satisfaction with continuing care in patients with first-ever stroke

Stroke recovery is a long-term process requiring persistent effort and cooperation from patients and families. However, most family members have limited knowledge about stroke rehabilitation, which affects recovery outcomes (21). The results of this study show that the experimental group had significantly higher disease-related knowledge awareness rates and satisfaction rates with continuing care compared to the control group, and the differences were statistically significant (*P* < 0.05).

Our personalized knowledge push system achieved 92% patient satisfaction, surpassing Wang et al.'s (2023) 89% using generic videos, highlighting the value of tailored health education (22).

This study created videos and graphics on stroke-related knowledge (medication, diet, exercise management, etc.) and uploaded them to the Continuing Care Management System. The nurse responsible delivered this content at discharge, allowing patients and families to access it conveniently via their mobile phones, ensuring timely knowledge acquisition, reinforcing memory, and improving patients’ self-awareness of the disease. The National Health Literacy Promotion Three-Year Action Plan (2024–2027) (23) issued by the National Health Commission provides directions for medical science popularization. Enhancing public health literacy is a key mission in the healthcare field under the “Healthy China” strategy. The research team also delivered various health education videos produced by the hospital's clinical departments for patients to watch, gradually deepening their disease understanding, improving their knowledge, attitudes, and practices, self-care abilities, and rehabilitation compliance (24), which positively impacted patient satisfaction.

### 4.4 Research limitation and future improvement plan

This study preliminarily validated the positive effects of information platform-based continuing care on discharged patients with first-episode stroke, but certain limitations remain. First, this was a single-center study, with samples all from the same hospital, resulting in a relatively limited sample size. Furthermore, convenience sampling was used, which may limit the external validity and generalizability of the results, potentially making them generalizable to a wider and more diverse patient population or different healthcare settings. Second, the follow-up period was short, only 3 months after discharge, limiting our assessment of the long-term effects of the intervention (e.g., neurological function recovery at 6 months, 1 year, or longer, complications, readmissions, and quality of life). Stroke rehabilitation is a long-term process, and short-term results may not fully reflect the sustained benefits of the intervention.

Given the aforementioned limitations, future research can be improved in the following ways:

1. Conduct multi-center, large-sample randomized controlled trials: Include patients from different regions and levels of medical institutions to improve the representativeness and extrapolation of the research results.

2. Extend the follow-up period: Design longer-term follow-up studies (e.g., 6 months, 1 year, 2 years) to evaluate the long-term efficacy, cost-effectiveness, and long-term impact on patients’ quality of life of this continuing care management system.

3. Explore the depth of the intervention mechanism: Future research can further analyze which specific functional modules of the platform (e.g., the specific content of health education, the timeliness of consultation, and the intervention of home care services) play a key role in improving various outcome indicators, thereby optimizing the intervention program.

4. Expand the scope of application: Consider applying this system model to the continuing care management of patients with other chronic diseases (e.g., coronary heart disease, diabetes, chronic obstructive pulmonary disease, etc.) to verify its cross-disease applicability and effectiveness.

## 5 Conclusion

This study constructed and implemented a continuing care intervention program for discharged patients with first-episode stroke based on an information platform. A randomized controlled trial involving 90 patients over a 3-month period demonstrated that adding a WeChat mini-program-based “Continuing Care Management System” intervention to routine continuing care significantly improved neurological function recovery (manifested as decreased mRS and NIHSS scores) and activities of daily living (ADL scores) in discharged patients. It effectively reduced the incidence of post-discharge complications (such as falls) and stroke-related readmissions, and significantly improved patients’ disease awareness and nursing service satisfaction. This confirms the positive value and application potential of an information-based continuing care model integrating online consultation and offline home care in promoting community and family rehabilitation for stroke patients.

However, this study has limitations due to its single-center design and short follow-up period. Therefore, future studies should further validate the effectiveness and universality of this system through multi-center, large-sample, and long-term research, and explore its application prospects in broader chronic disease management, thereby providing stronger evidence to support smart healthcare and continuous healthcare services under the “Healthy China” strategy.

## Data Availability

The original contributions presented in the study are included in the article/Supplementary Material, further inquiries can be directed to the corresponding author.
